# Sulforaphane-cysteine inhibited migration and invasion via enhancing mitophagosome fusion to lysosome in human glioblastoma cells

**DOI:** 10.1038/s41419-020-03024-5

**Published:** 2020-10-01

**Authors:** Yan Zhou, Yalin Wang, Sai Wu, Yuting Yan, Yabin Hu, Zhongnan Zheng, Juntao Li, Wei Wu

**Affiliations:** 1 Department of Biochemistry and Molecular Biology, School of Basic Medical Sciences, Capital Medical University, Beijing, 100069 China; 2 Beijing Key Laboratory for Invasion and Metastasis, Capital Medical University, No. 10, Xitoutiao, You An Men Wai Ave., Feng Tai District, Beijing, 100069 China

**Keywords:** CNS cancer, Cell invasion

## Abstract

Here we uncovered the involved subcellular mechanisms that sulforaphane-cysteine (SFN-Cys) inhibited invasion in human glioblastoma (GBM). SFN-Cys significantly upregulated 45 and downregulated 14 microtubule-, mitophagy-, and invasion-associated proteins in GBM cells via HPLC–MS/MS and GEO ontology analysis; SFN-Cys disrupted microtubule by ERK1/2 phosphorylation-mediated downregulation of α-tubulin and Stathmin-1 leading to the inhibition of cell migration and invasion; SFN-Cys downregulated invasion-associated Claudin-5 and S100A4, and decreased the interaction of α-tubulin to Claudin-5. Knockdown of Claudin-5 and S100A4 significantly reduced the migration and invasion. Besides, SFN-Cys lowered the expressions of α-tubulin-mediated mitophagy-associated proteins Bnip3 and Nix. Transmission electron microscopy showed more membrane-deficient mitochondria and accumulated mitophagosomes in GBM cells, and mitochondria fusion might be downregulated because that SFN-Cys downregulated mitochondrial fusion protein OPA1. SFN-Cys increased the colocalization and interplay of LC3 to lysosomal membrane-associated protein LAMP1, aggravating the fusion of mitophagosome to lysosome. Nevertheless, SFN-Cys inhibited the lysosomal proteolytic capacity causing LC3II/LC3I elevation but autophagy substrate SQSTM1/p62 was not changed, mitophagosome accumulation, and the inhibition of migration and invasion in GBM cells. These results will help us develop high-efficiency and low-toxicity anticancer drugs to inhibit migration and invasion in GBM.

## Introduction

High invasion in human glioblastoma (GBM) is the key reason to cause low survival and bad outcome^1^. Plant-derived sulforaphane (SFN) induced apoptosis and inhibited invasion by ERK1/2 phosphorylation in various cancers^2–5^. Sulforaphane-cysteine (SFN-Cys), one of the SFN metabolites, has great potential to penetrate blood–brain barrier and a longer half-life in the brain tissue than SFN^6^. We previously reported that SFN-Cys inhibited migration and invasion via microtubule-mediated Claudins dysfunction in human non-small cell lung cancer (NSCLC) cells^7^. We also revealed that SFN-Cys suppressed invasion via downregulating Galectin-1 in human prostate cancer^8^. Here at subcellular levels we will determine how SFN-Cys inhibited migration and invasion in human GBM to establish a high-efficiency anticancer chemotherapy.

Microtubule plays a vital role in cell motility, migration, and invasion of tumor cells via assembly and disassembly of α-tubulin and β-tubulin complex^9^. Actins and tubulins form highly dynamic polymers that are capable of organizing cytoplasmic organelles, determining cell shape and polarity and promoting cell–cell and cell–matrix adhesions through their interactions with cadherins and integrins, respectively^10^. Studies showed that microtubule-associated protein Stathmin-1 binds to tubulins promoting the depolymerization of microtubule^11^, while the inhibition of stathmin expression significantly reduced transendothelial migration in neuroblastoma cells^12^. Just recently, we found that SFN-Cys triggered microtubule disruption via decreasing Stathmin-1 leading to microtubule depolymerization in human prostate cancer^13^. Therefore, we predicted that SFN-Cys might inhibit migration and invasion via regulating Stathmin-1-assoicated microtubule disruption in human GBM cells.

Invasion-associated proteins, membrane proteins, or adhesion molecules might contribute to tumor migration and invasion. Claudin-5 is one of the main components of tight junctions for cancer cells to connect adjacent cells^14^. Studies demonstrated that cell proliferation and invasion were reduced after silencing Claudin-5^15^. We previously reported that sustained phosphorylation of ERK1/2 contributed to invasion inhibition^5,8^. SFN promoted the phosphorylation of ERK1/2 downregulating matrix metalloproteinase MMP-2 leading to invasion inhibition in GBM^4^. Claudin-5 was determined to interact with the MT1 matrix metalloproteinase increasing cell migration and invasion via degradation of the extracellular matrix^16,17^. We found that knockdown of α-tubulin downregulated Claudins and inhibited migration and invasion, indicating that microtubule disruption contributed to invasive inhibition in NSCLC cells^7^. The cytosolic C-terminal domain of Claudin-5 contains a PDZ-binding domain which binds ZO-1, ZO-2, and ZO-3 proteins, consequently binding to the microtubules^18^. Besides, Claudin-11 was proved to interact with α-tubulin promoting cell migration, indicating that microtubule might function as a mediator to regulate Claudins signaling, autophagy and invasion^19^. SFN-Cys might downregulate a dozen of proteins via phosphorylated ERK1/2-mediated signals^7,8,13^. S100A4 is a member of the S100 protein family, which is associated with cell motility^20^. S100A4 is known to promote invasion and metastasis in human GBM cells^21^; knockdown of S100A4 reduced tumorigenesis and metastasis in tumor cells^22^. Therefore, here it is necessary to investigate whether SFN-Cys inhibits invasion via regulating S100A4 and Claudin-5.

Autophagy is an intracellular degradation process maintaining cell homeostasis by clearing damaged organelles and proteins; microtubule played a critical role in regulating such a process. At autophagy, microtubule-associated protein light chain 3 II (LC3II) was recruited onto autophagosomal membrane, moving along microtubule to fuse autophagosome to lysosome for degradation, and the level of autophagic substrate SQSTM1/p62 (sequestosome 1) decreased^23,24^. We previously reported that another SFN metabolite, sulforaphane-N-acetyl-cysteine (SFN-NAC) induced microtubule disruption promoting the formation and accumulation of autophagosome leading to apoptosis in NSCLC cells^25^. Besides, both SFN-Cys and SFN-NAC also inhibited migration and invasion via interfering autolysosome formation in NSCLC cells^13^. However, at subcellular level, it is not clear how SFN and its metabolites regulate mitophagy inhibiting cell migration and invasion.

Mitophagy maintains cell homeostasis via specifically degrading abnormal mitochondria. Both Bnip3 (Bcl-2/adenovirus E1B 19-kDa-interacting protein 3) and Nix (BNIP3L) interact directly with LC3 to promote mitophagy^26^. Bnip3 is a mitochondrial BH3-only protein that contributes to mitophagy^26^. Its receptor Nix directly interacts with LC3, and mediated the subsequent binding and sequestration of mitochondrial proteins to autophagosomes^25^. Besides, Bnip3 increases the rate of mitophagy in response to hypoxia promoting tumor progression to metastasis^27^. In the present study, we will investigate whether SFN-Cys regulates mitochondrial autophagy by changing microtubule dynamics, leading to the inhibition of migration and invasion in GBM.

Taken together, SFN-Cys might cause microtubule disruption, regulate mitophagy and the expression of S100A4 and Claudin-5 inhibiting migration and invasion in GBM cells; the investigation of the underlying mechanisms will help us design brand-new, effective and safe drugs to treat invasive cancers.

## Materials and methods

### Reagents and antibodies

D, L-sulforaphane-L-cysteine (SFN-Cys) (sc-207499) was purchased from Santa Cruz Biotechnology (Dallas, Texas, USA). Anti-p-ERK1/2 (9101S), anti-ERK1/2 (9102S), anti-LAMP1 (15665), and PD98059 (9900S) were obtained from Cell Signaling Technology (Danvers, MA, USA). Anti-Stathmin-1 (D260545) and anti-pStathmin-1 (Ser 25) (D155102) were purchased from Sangon Biotech (Shanghai, China). Anti-Claudin-5 (ab131259), anti-S100A4 (ab124805), anti-Bnip3 (ab109362), anti-Nix (ab109414), anti- anti-SQSTM1/p62 (ab207305), and anti-OPA1 (ab157457) were purchased from Abcam (Cambridge, UK). Matrigel basement membrane matrix (356234) was bought from BD Biosciences (Franklin Lakes, NJ, USA). Mitochondrial Isolation Kit (ProteinExt^TM^) (DE401-01) was purchased from TransGen Biotech (Beijing, China). Mitophagy inducer carbonyl cyanide 3-chlorophenylhydrazone (CCCP) (C2759) was purchased from Sigma-Aldrich (St-Louis, Missouri, USA). Bafilomycin A1 (Baf-A1) (S1413) was purchased from Selleck Chemicals (Houston, DX, USA). Chloroquine diphosphate (T0194) was purchased from Topscience (Shanghai, China). Anti-α-tubulin (11224-1-AP) and anti-β-actin (60008-1-Ig) were ordered from Proteintech (Wuhan, Hubei, China).

### Cell culture

Human GBM U87MG cell line was purchased from the Cell Resource Center, Peking Union Medical College (CRC/PUMC) and U373MG cell line was purchased from American Type Culture Collection (ATCC, USA). The species origin was confirmed with PCR, and the cell identity was authenticated with STR profiling by FuHeng Biology (Shanghai, China). Cells were cultured in DMEM/HIGH glucose culture medium (SH30243.01B, Hyclone, Logan, USA) supplemented with 10% fetal bovine serum (FBS) (SE100-B, Vistech, Australia), 100 U/ml penicillin and 100 U/ml streptomycin solution (P1400-100) (Solarbio, Beijing, China) in a standard humidified incubator with 5% CO_2_ at 37 °C. Generally, the cultured cells in logarithmic phase were randomly allocated to group and treated by SFN-Cys and/or other stimuli in each assay.

### High performance liquid chromatography–mass spectrometry/mass spectrometry (HPLC–MS/MS)

For the glycopeptide identification, HPLC–MS/MS analysis was performed via an Orbitrap Fusion Lumos mass spectrometer (Thermo Scientific, USA) equipped with a nanoelectrospray ionization source and an EASY-nLC 1000 liquid chromatography system (Thermo Scientific, USA). The samples were dissolved in 0.1% FA and separated on a capillary column (150 µm id ×120 mm) packed with C18 (1.9 µm, 100 Å,) at a flow rate of 600 nl/min. The mobile phase consisted of 0.1% formic acid in water (A) and 0.1% formic acid in acetonitrile (ACN) (B). Mobile phase A (99.9% water/0.1% FA) and mobile phase B (99.9% ACN/0.1% FA) were used, and the elution gradient used was from 6 to 32% mobile phase B for 78 min. Data acquisition was performed by the data-dependent mode. The recognized peptides were searched from UniProt Knowledgebase (https://www.uniprot.org/). The threshold of 1.5-fold or 0.66-fold changes (*p* < 0.05) was defined based on the ratio of values in the treated group versus control group as an upregulation or downregulation. The control or SFN-Cys-treated group was performed in triplicate.

### Western blot

Total cellular protein lysates were harvested and lysed in RIPA lysis buffer (C1053, Applygen, Beijing, China) with a protease inhibitor cocktail (04693132001, Roche, Shanghai, China). Protein concentration was evaluated by the BCA protein assay kit (P1511, Applygen, Bejing, China). Equal amounts of total proteins were separated with SDS-PAGE (12%) for the detection of ERK1/2, p-ERK1/2, Stathmin-1, pStathmin-1, α-tubulin, Claudin-5, S100A4, Bnip3, Nix, SQSTM1/p62, and OPA1. β-actin was used as the protein loading control. After SDS-PAGE, the proteins in the gels were transferred to BioTrace nitrocellulose membranes (66485, Pall, New York, USA) with 7.5% bovine serum albumin (BSA) blockage in Tris-buffered saline (pH7.4, 20 mM Tris-HCl, 150 mM NaCl), and then incubated with afore-mentioned primary antibodies overnight at 4 °C. The membranes were incubated with IRDye-labeled goat anti-mouse or goat anti-rabbit IgG (LI-COR, Lincoln, NE, USA) at the room temperature for 1 h. Finally, the protein bands were scanned by the LI-COR Odyssey system (LI-COR, Lincoln, NE, USA). At least three independent experiments were performed. The grayscale of the protein bands was calculated and normalized by densitometry of β-actin signal by Image J software.

### RNA interference

Claudin-5 siRNA (5′-UUCAUUCCGUCUGUUAAGGTT-3′) ^28^ and designed S100A4 siRNA (5′-UACUUGUGGAAGGUGACACCAUTT-3′) as well as control siRNA (5′-ACGUGACACGUUCGGAGAATT-3′) were synthesized to silence Claudin-5 and S100A4 (Gene Pharma, Shanghai, China). Cells were plated in six-well plates and cultured for 24 h. Then the siRNAs (30 pmol/well) with Lipofectamine^TM^ RNAiMAX (13778075, Invitrogen, CA, USA) were transfected into cells when cells reached ~80% confluency. After the transfected cells were cultured for more than 24 h, cells were randomly divided into two plates and treated with or without SFN-Cys at 60–70% confluency.

### Scratch assay

Wound scratch healing assay was performed to detect the capacity of cell migration. Cells were seeded at a density of 2 × 10^6^ cells per well in a six-well plate and cultured overnight. Three parallel thin “wounds” and one vertical “wound” were scratched by a pipette tip when cells reach ~100% confluency. The cells were washed with phosphate-buffered saline (PBS) and treated by serial concentrations of SFN-Cys with serum-free medium for 24 h. The images were captured by a phase-contrast microscope at 0 and 24 h, and the relative wound areas were calculated by the Image-pro plus 6.0. The migrated cells were observed in at least five to six randomly selected fields per well under microscope. The ratio of wound area in 0 h vs. 24 h represented the inhibitory capacity of migration and invasion by SFN-Cys. At least three independent experiments were performed.

### Invasion assay

The transwell chambers placed into a 24-well plate were rehydrated at 37 °C for 1 h before seeding cells. Matrigel basement membrane matrix was diluted with serum-free DMEM medium to 2 mg/ml and then plated onto transwell chamber. A total of 2 × 10^4^ cells were added to the upper chamber with serum-free DMEM medium and 500 µl DMEM medium containing 10% FBS was added to the lower chamber. After treated with different concentrations of SFN-Cys for 24 h, the cells were fixed with 100% methanol for 20 min and then stained with 0.5% crystal violet solution for 30 min. The cells were washed three times with PBS and the cells on the top of upper chamber were removed gently with a cotton swab. The invasive cells were observed in at least five to six randomly selected fields per well under microscope. The data were analyzed by Image J software. At least three independent experiments were performed.

### Bioinformatics analysis

We searched the GEPIA (Gene Expression Profiling Interactive Analysis) Database to find the possible correlations between survival rate and expression to microtubule related proteins. GEPIA is a public database newly developed by the Chinese for cancer and normal gene expression profiling from 9736 tumors and 8587 normal samples. These results with statistical significance will be recorded (*p* < 0.05)^29^. We also searched the version 10.5 of STRING database to get the data for protein correlation and drew the Protein–Protein Interaction Map using Cytoscape software^30^.

### Immunofluorescence staining and confocal microscopy

Cells were seeded in 35 mm cover glass-bottom dishes at a density of 1 × 10^5^ cells/dish and incubated for 24 h, then treated with 20 µM SFN-Cys and/or 10 μM CCCP. The cells were fixed with 4% paraformaldehyde for 15 min. After washed by PBST (PBS with Tween-20), the cells were permeabilized with −20 °C methanol for 10 min at the room temperature. After blocking with PBS containing 1% BSA and 0.1% Triton X-100 for 1 h, the cells were incubated with anti-α-tubulin or anti-LAMP1 and anti-LC3 overnight at 4 °C. After washed three times with PBST, the cells were incubated with the fluorescence-labeled secondary antibody for 1 h at the room temperature, followed with adding fluorescent mounting medium with DAPI (4,6-diamidino-2-phenylindole) (DAPI) (ZLI-9557, ZSGB-Bio, China). All the measurements were performed by a researcher who was blinded to group allocation with a laser scanning confocal microscope (Olympus FV1000, Olympus, Japan). The Pearson’s_Rr was calculated by Image J software.

### Co-immunoprecipitation

Cells were plated at a density of 5 × 10^6^ cells/dish and treated with 20 μM SFN-Cys for 24 h. After washed with ice-cold PBS, cells were lysed on ice via Nondenaturing Lysis Buffer (C1050, Applygen, Beijing, China) with protease inhibitor cocktail (04693132001, Roche, Shanghai, China). After total proteins were quantified, equal amount of proteins were incubated with the fixed primary antibodies overnight at 4 °C. Then, the complexes were co-incubated with protein A/G agarose (sc-2003, SantaCruz, Dallas, Texas, USA) rotationally for 3 h at 4 °C and the proteins were isolated from the beads by centrifuging and boiled for 5 min. Western blot was used to recognize the conjugated proteins.

### Transmission electron microscopy

Cells were incubated with or without 20 μM SFN-Cys for 24 h, then harvested and fixed with 3% glutaraldehyde at 4 °C for 2 h. After washed with PBS for three times, samples were fixed in 1% osmium tetroxide for 1 h. Then samples were dehydrated through a graded ethanol series and embedded in a 1:1 mixture of acetone and Epon812 resin for 30 min. The samples were then infiltrated 2 h in Epon812. Ultrathin sections were cut with a knife and placed on 200-mesh copper grids and stained with Uranium acetate for 30 min and Lead nitrate for 20 min. All the measurements were performed by a researcher who was blinded to group allocation with a transmission electron microscope (JEM-1400Plus, JEOL, Japan).

### Mitochondrial protein extraction

Cells (2 × 10^7^) were treated with or without 20 μM SFN-Cys for 24 h, then were harvested by centrifugation at 1000 × *g* for 3 min. Mitochondrial Isolation Kit (ProteinExt^TM^) (DE401-01, TransGen, Beijing, China) was used to isolate mitochondrial proteins by the manufacturer’s instructions. Cells were lysed with 400 μl MIB I and 5 μl MIB II, respectively, for 2 and 5 min on ice with severe shake. After mixed with 400 μl MIB III, cells were centrifuged at 700 × *g* at 4 °C for 10 min. The supernatant was centrifuged again at 12,000 × *g* at 4 °C for 15 min; the harvested supernatant contained the cytosolic proteins. Then the obtained pellet was suspended with 500 μl MIB III and was centrifuged at 12,000 × *g* at 4 °C for 15 min. The final pellet was lysed to prepare the mitochondrial proteins.

### Autophagy and mitophagy assay

Western blot analysis was used to detect the LC3II, the indicator of autophagosome formation. Mitophagy inducer CCCP (10 μM) was used to induce mitophagy to test the migration and invasion in tumor cells. Bafilomycin A1 (Baf-A1, 50 nM) or chloroquine (25 μM) was used to block the fusion of autophagosome/mitophagosome to lysosome and inhibit the lysosomal activity, respectively. TEM was performed to observe the subcellular morphology.

### Statistical analysis

All data represent at least three independent experiments. Differences among groups and treatments for all in vitro experiments were determined by two-tailed Student’s *t* test in GraphPad Prism and Excel. Bar graph represents quantification data as means ± SD from at least three biological replicates (*n* ≥ 3). *P* values of less than 0.05 represented statistical significance.

## Results

### SFN-Cys regulated proteomic expressions and inhibited migration and invasion in a dose-dependent manner

**Fig. 1:**
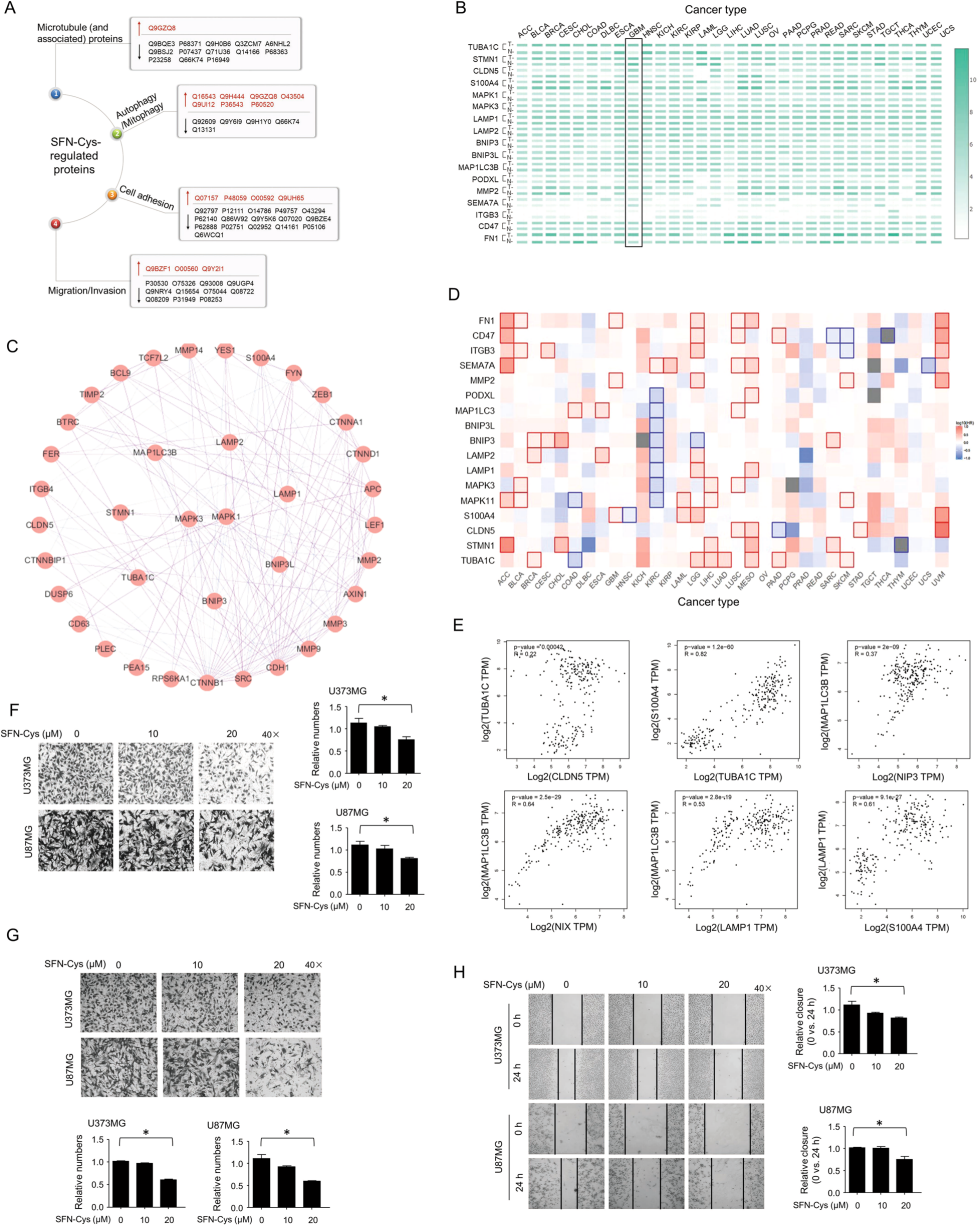
SFN-Cys regulated proteomic expression and inhibited cell migration and invasion in U373MG and U87MG cells. **a** The SFN-Cys-regulated protein expressions in U87MG cells were identified by HPLC–MS/MS. Red, upregulation; black, downregulation. **b** Gene Expression Profile was performed in predicted SFN-Cys-targeted proteins in either tumor (T) or normal (N) tissue among different cancers by GEPIA. **c** The Protein–Protein Interaction network in SFN-Cys-targeted proteins was predicted by String server. **d** The Overall Survival Map of predicted SFN-Cys-targeted proteins in different cancers by GEPIA. **e** The Correlation Analysis among SFN-Cys-targeted TUBA1C, CLDN5, S100A4, MAP1LC3B, Bnip3, Nix, and LAMP1 in GBM by GEPIA server (*p* < 0.05). Migration assay without matrigel (**f**) or invasion assay with matrigel (**g**) was performed separately to detect the numbers of migratory or invasive cells in U87MG and U373MG cells after cells were treated with 0, 10, and 20 μM SFN-Cys for 24 h. **h** The area covered by migratory cells was recorded by Leica DMIRB microscope at ×40 magnification at 0 and 24 h. The relative closure (0 h vs. 24 h) was measured by Image J. At least three independent experiments were performed. *p < 0.05. The values are expressed as means ± SD (*n* ≥ 3).

SFN-Cys upregulated 45 and downregulated 14 protein expressions in U87MG cells via HPLC–MS/MS analysis; these proteins were associated with microtubule, mitophagy, migration, and invasion (Fig. 1a) (Supplementary Table 1). Of these, gene expression analysis by GEPIA server showed that the expressions of TUBA1C, S100A4, CLDN5 (Claudin-5), MAPK1, LAMP1, LAMP2, BNIP3L (Nix), MAP1LC3B (LC3), PODXL, MMP-2, ITGB3, CD47, and FN1 were upregulated, while SEMA7A expression was downregulated in GBM tissues (Fig. 1b). Protein–Protein Interaction network was done and the results showed that both CLDN5 and S100A4 might interact with invasion-associated matrix metalloproteinase MMPs (Fig. 1c). Besides, TUBA1C, STMN1, BNIP3, BNIP3L, MAP1LC3B, LAMP1, LAMP2, MAPK1, and MAPK3 might interact one another (Fig. 1c). Survival analysis showed that the expressions of S100A4, FN1, and MMP-2 were negatively correlated to over-survival significantly in GBM and Brain Lower Grade Glioma (Fig. 1d). Further, the data obtained from Gene Correlation Analysis showed that the expression of TUBA1C was positively correlated to the expression of CLDN5 and S100A4; MAP1LC3B expression was positively correlated to the expressions of Bnip3, Nix and LAMP1; LAMP1 expression was positively correlated to S100A4 expression in GBM (Fig. 1e). We found that 20 μM SFN-Cys inhibited migration and invasion without inducing apoptosis in U373MG and U87MG cells; this is the optimal dose for cell motility assay. It was shown that the number of migrant cells was significantly decreased with the increase of SFN-Cys dose by migration assay (Fig. 1f). Similarly, the number of invasive cells was significantly decreased with the increase of SFN-Cys dose by invasion assay (Fig. 1g). Also, the relative wound closures (0 h vs. 24 h) were decreased with the increase of SFN-Cys dose by scratch assay (Fig. 1h). These indicated that SFN-Cys inhibited migration and invasion in U373MG and U87MG cells in a dose-dependent manner.

### SFN-Cys upregulated phosphorylated ERK1/2 inhibiting migration and invasion

**Fig. 2:**
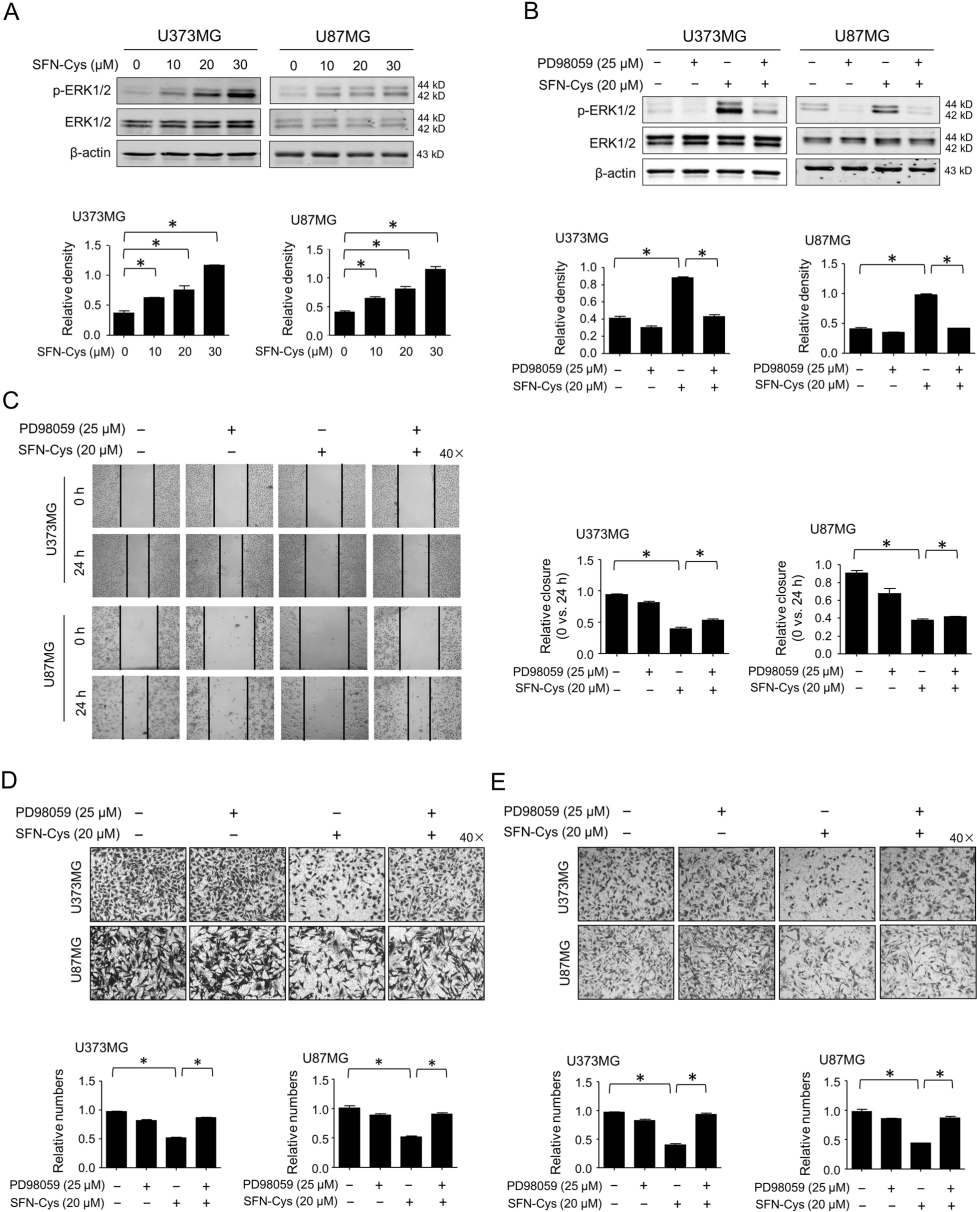
SFN-Cys inhibited cell migration and invasion via sustained activation of ERK1/2 in U373MG and U87MG cells. The expressions of p-ERK1/2 and ERK1/2 were detected by western blot with the treatment of 0, 10, 20, and 30 μM SFN-Cys for 24 h (**a**) or with the treatment of 20 μM SFN-Cys and/or 25 μM PD98059 for 24 h (**b**). The migratory ability was examined by scratch assay (**c**) or by migration assay (**d**) with the treatment of 20 μM SFN-Cys and/or 25 μM PD98059 for 24 h. **e** The invasion ability was detected by invasion assay with the treatment of 20 μM SFN-Cys and/or 25 μM PD98059 for 24 h. β-actin was used to be the loading control. At least three independent experiments were performed. *p < 0.05. Data were shown as means ± SD (*n* ≥ 3).

We have uncovered that SFN-Cys inhibited invasion in a couple of cancer types including NSCLC and prostate cancer^5,8^. Here, results showed that SFN-Cys induced the phosphorylation of ERK1/2 (Thr202/Tyr204) in a dose-dependent manner (Fig. 2a). However, phosphorylated ERK1/2 inhibitor PD98059 reversed the SFN-Cys-induced phosphorylation of ERK1/2 (Fig. 2b). Then, PD98059 was used to determine the linkage of SFN-Cys-regulated ERK1/2 signaling to cell migration and invasion in GBM cells. Results showed that the relative wound closures (0 h vs. 24 h) were decreased after cells were treated with 20 μM SFN-Cys by scratch assay, while such reduction was reversed by PD98059 (Fig. 2c). The numbers of migrant cells were significantly decreased after cells were treated with 20 μM SFN-Cys by migration assay, while such reduction was reversed by PD98059 (Fig. 2d). Likewise, the invasive cells were decreased with the treatment of 20 μM SFN-Cys by invasion assay in matrigel, which was reversed by PD98059 (Fig. 2e). These results indicated that SFN-Cys mediated the phosphorylation of ERK1/2 inhibiting the migration and invasion in human GBM cells.

### SFN-Cys-mediated ERK1/2 phosphorylation downregulated α-tubulin and Stathmin-1, upregulated phosphorylated Stathmin-1 triggering microtubule disruption

**Fig. 3:**
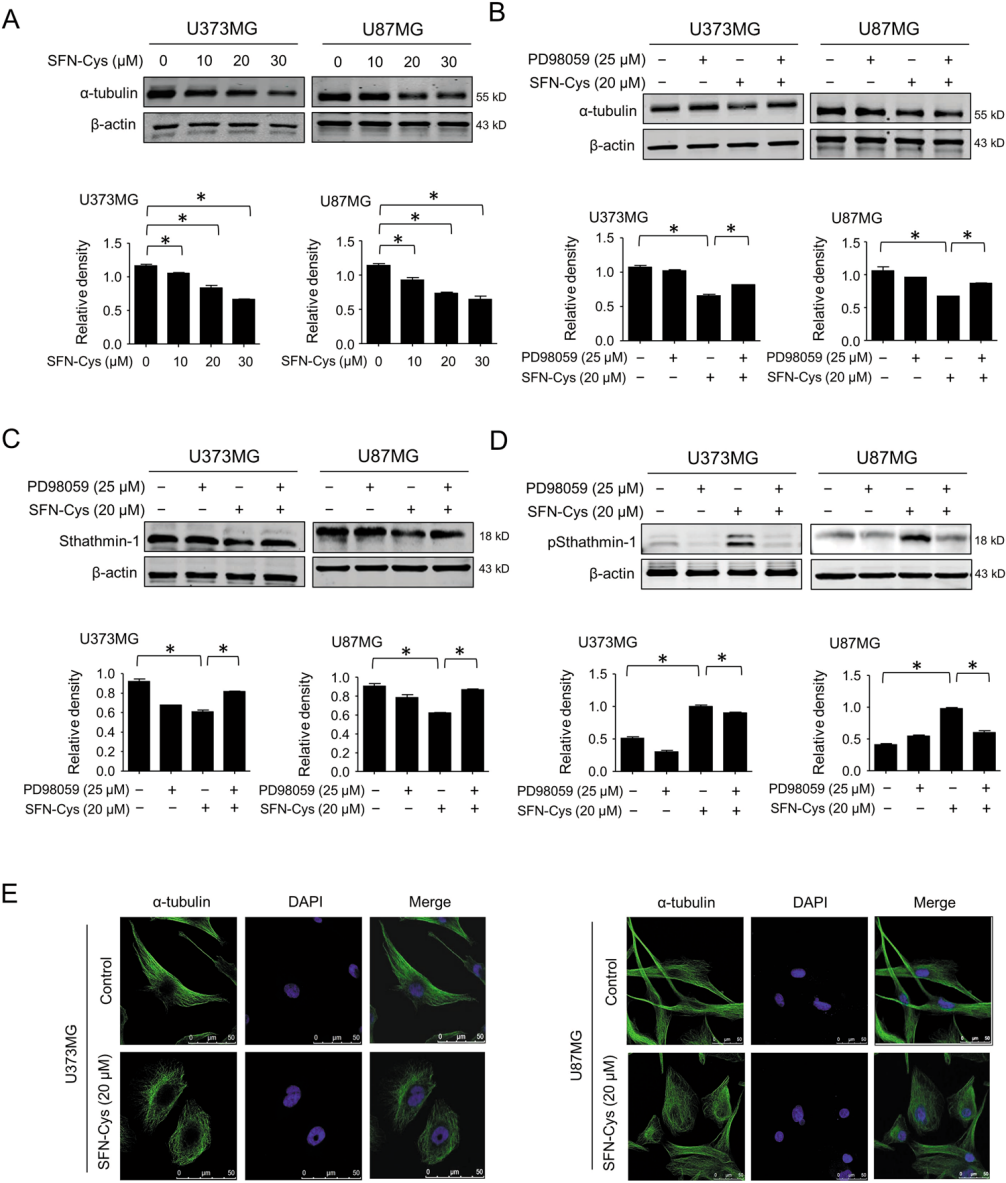
SFN-Cys changed microtubule morphology and downregulated α-tubulin via sustained activation of ERK1/2. The expression of α-tubulin was detected by western blot with the treatment of 0, 10, 20, and 30 μM SFN-Cys for 24 h (**a**) or with the treatment of 20 μM SFN-Cys and/or 25 μM PD98059 for 24 h (**b**). The expression of Stathmin-1 (**c**) or pStathmin-1 (Ser 25) (**d**) was detected by western blot with the treatment of 20 μM SFN-Cys and/or 25 μM PD98059 for 24 h. **e** Cells were treated with or without 20 μM SFN-Cys and the microtubule morphology was observed by immunofluorescence and confocal microcopy. Scale bar = 50 μm. β-actin was used to be the loading control. At least three independent experiments were performed. *p < 0.05. Data were shown as means ± SD (*n* ≥ 3).

We reported that SFN-Cys downregulated α-tubulin in the previous studies^13^. Results showed that SFN-Cys downregulated α-tubulin in a dose-dependent manner in GBM cells (Fig. 3a), while such downregulation was reversed by PD98059 (25 μM) (Fig. 3b), indicating that SFN-Cys downregulated α-tubulin by the phosphorylation of ERK1/2. Besides, SFN-Cys downregulated Stathmin-1 and upregulated pStathmin-1 (Ser 25), and these results were reversed by PD98059 (Fig. 3c, d). These data indicated that SFN-Cys modulated Stathmin-1 and pStathmin-1 (Ser 25) via ERK1/2 phosphorylation in GBM cells. Meanwhile, we observed the morphological changes of microtubule by immunofluorescence assay. The results showed that microtubules were filamentous and distributed homogeneously in the control cells, while the cell tentacles disappeared and the microtubules were disrupted and aggregated in SFN-Cys-treated cells (Fig. 3e).

### SFN-Cys inhibited migration and invasion by downregulating Claudin-5 and S100A4

**Fig. 4:**
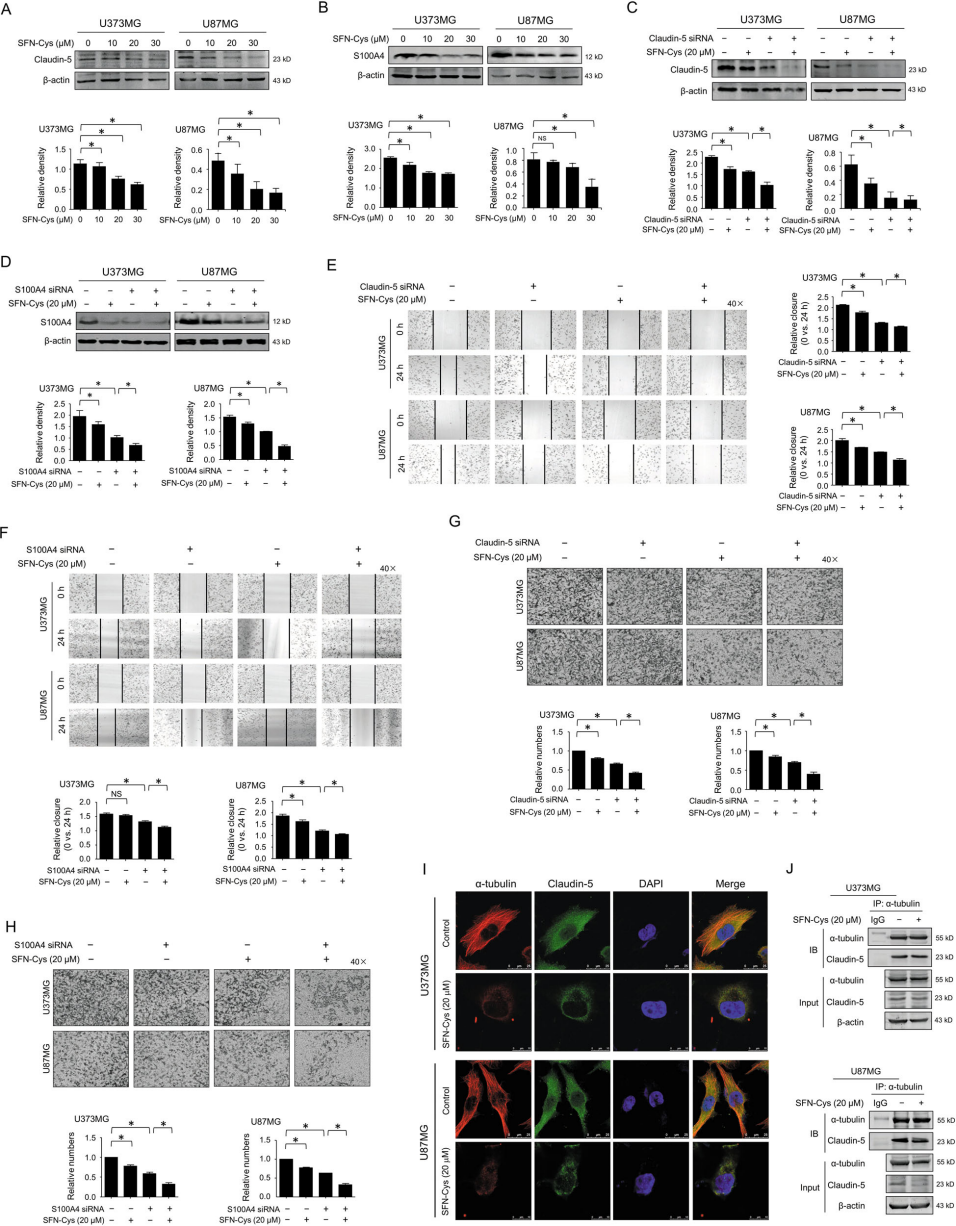
SFN-Cys inhibited migration and invasion by downregulating S100A4 and α-tubulin-mediated Claudin-5. The expression of Claudin-5 (**a**) or S100A4 (**b**) was detected by western blot with the treatment of 0, 10, 20, and 30 μM SFN-Cys for 24 h. The expression of Claudin-5 (**c**) or S100A4 (**d**) was detected by western blot with the treatment of 20 μM SFN-Cys and/or knockdown of Claudin-5 (**c**) or S100A4 (**d**) for 24 h. Scratch assay was performed to evaluate the migration ability after knockdown of Claudin-5 (**e**) or S100A4 (**f**) with or without 20 μM SFN-Cys treatment for 24 h. Invasion assay was performed to evaluate the invasion ability after knockdown of Claudin-5 (**g**) or S100A4 (**h**) with or without the treatment of 20 μM SFN-Cys for 24 h. **i** Immunofluorescence staining showed the cellular colocalization of Claudin-5 to α-tubulin and the microtubule morphology with the treatment of 20 μM SFN-Cys. Red: stained α-tubulin; green: stained Claudin-5; blue: DAPI-stained DNA; scale bar = 25 μm. **j** Cells were treated with 20 μM SFN-Cys for 24 h. The interplay between Claudin-5 and α-tubulin was detected by co-immunoprecipitation (Co-IP). β-actin was used to be the loading control. At least three independent experiments were performed. *p < 0.05; NS no significance. Data were shown as means ± SD (*n* ≥ 3).

It was shown that Claudin-5 and S100A4 were downregulated after cells were treated with 0, 10, 20, 30 μM SFN-Cys for 24 h by western blot in GBM cells (Fig. 4a, b). Claudin-5 and S100A4 siRNAs were successfully used to silence Claudin-5 and S100A4 (Fig. 4c, d). By scratch assay, SFN-Cys-induced decrease of migratory capacity was significantly aggravated by either Claudin-5 or S100A4 silencing (Fig. 4e, f). Further, invasion assay showed that SFN-Cys-induced decrease of invasion capacity was also significantly exacerbated by either Claudin-5 or S100A4 silencing (Fig. 4g, h). These results indicated that SFN-Cys inhibited migration and invasion via downregulating Claudin-5 and S100A4 in human GBM cells. Immunofluorescence and confocal microscopy results showed that SFN-Cys suppressed the colocalization of Claudin-5 to α-tubulin (Fig. 4i). Meanwhile, by co-immunoprecipitation we found that SFN-Cys reduced the interaction of Claudin-5 to α-tubulin (Fig. 4j). Therefore, SFN-Cys might reduce the microtubule-associated Claudin-5 causing the inhibition of migration and invasion in human GBM cells.

### SFN-Cys caused mitophagosome accumulation by disrupting microtubules

**Fig. 5:**
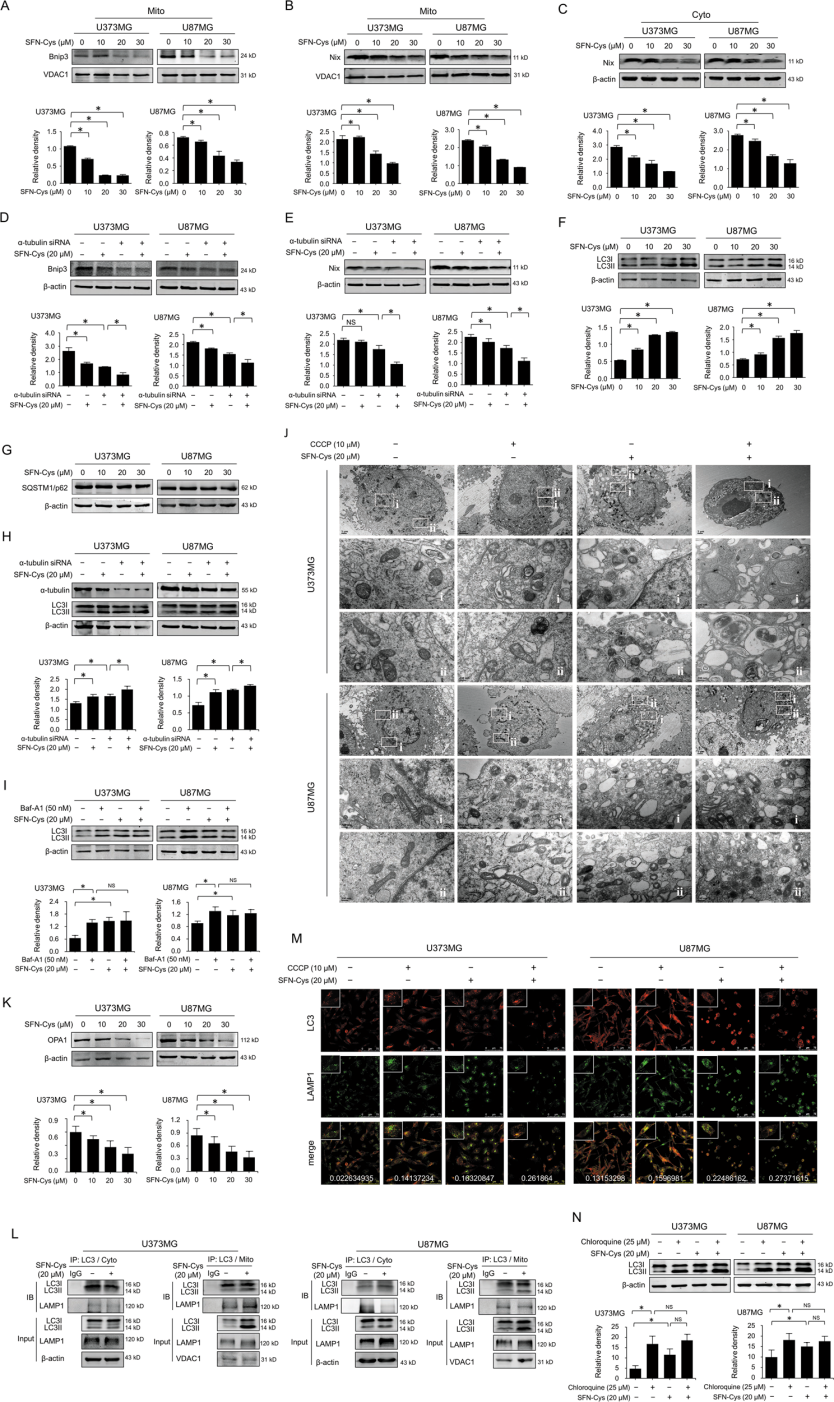
SFN-Cys inhibited mitochondrial autophagy by downregulating Bnip3 and Nix and microtubule disruption. The expression of Bnip3 in mitochondria (**a**) or Nix in mitochondria (**b**) and in cytosol (**c**) was detected by western blot with the treatment of 0, 10, 20, and 30 μM SFN-Cys for 24 h. The expression of Bnip3 (**d**) or Nix (**e**) was detected by western blot after knockdown of α-tubulin with/without the treatment of 20 μM SFN-Cys for 24 h. The expressions of LC3I and LC3II (**f**) and SQSTM1/p62 (**g**) were detected by western blot with the treatment of 0, 10, 20, and 30 μM SFN-Cys for 24 h. **h** The expressions of LC3I and LC3II were detected by western blot after knockdown of α-tubulin with/without the treatment of 20 μM SFN-Cys for 24 h. **i** The expressions of LC3I and LC3II were detected by western blot with the treatment of 20 μM SFN-Cys and/or 50 nM Bafilomycin A1 for 24 h. **j** Subcellular structures in U373MG and U87MG cells were viewed by TEM with 20 μM SFN-Cys and/or 10 μM CCCP treatment. Scale bar = 1 and 0.2 μm of magnification; “i” and “ii” in the lower panels matched the two local amplifications of the upper panel in each treated group. **k** The expression of OPA1 was detected by western blot with the treatment of 0, 10, 20, and 30 μM SFN-Cys for 24 h. The interaction of LC3 to LAMP1 was determined by co-IP both in cytosol and mitochondria treated with 20 μM SFN-Cys (**l**) or by confocal microcopy with the treatment of 20 μM SFN-Cys and/or 10 μM CCCP (**m**). Red: LC3, green: LAMP1, blue: nuclei. Scale bar = 75 μm. The local amplification was framed in each image. The colocalization was evaluated by Pearson’s_Rr recorded on the merged images. **n** The expression of LC3I and LC3II was detected by western blot with the treatment of 20 μM SFN-Cys and/or 25 μM chloroquine for 24 h. β-actin and VDAC1 were used to be the loading control of cytosolic/total and mitochondrial proteins. At least three independent experiments were performed. *p < 0.05; NS no significance. Data were shown as means ± SD (*n* ≥ 3).

Bnip3 and Nix are the mitophagy receptors that interact with LC3 promoting the sequestration of mitochondria into the isolation membrane. Here, we found that Bnip3 only presented in mitochondria (data not shown), and SFN-Cys decreased the expression of mitochondrial Bnip3 in a dose-dependent manner (Fig. 5a). Nevertheless, Nix was expressed both in mitochondria and cytoplasm, and SFN-Cys also downregulated both mitochondrial and cytosolic Nix in a dose-dependent manner (Fig. 5b, c). The expression of both Bnip3 and Nix were downregulated after knockdown of α-tubulin via RNA interference (Fig. 5d, e). These data indicated that SFN-Cys decreased α-tubulin causing the downregulation of Bnip3 and Nix, possibly inhibiting the formation of mitochondrial autophagosomes and selective clearance of mitochondria. However, SFN-Cys elevated the ratio of LC3II and LC3I (LC3II/LC3I) in a dose-dependent manner in both U373MG and U87MG cells (Fig. 5f), while unaffected the level of SQSTM1/p62 (Fig. 5g). SQSTM1/p62 is an autophagy substrate, and the reduction of SQSTM1/p62 was a marker of autophagy activation. Generally, SQSTM1/p62 is degraded in a normal autophagic process; otherwise the level of SQSTM1/p62 is not changed. These results implied that SFN-Cys might block the autophagic flux and inhibit the degradation of autophagosomes. Besides, SFN-Cys-mediated upregulation of LC3II/LC3I was aggravated after α-tubulin was silenced (Fig. 5h), suggesting that SFN-Cys decreased α-tubulin level triggering the accumulation of autophagosomes. Bafilomycin A1 (Baf-A1) is a vacuole H ^+^-ATPase inhibitor to inhibit the fusion of autophagosome to lysosome. Results showed that Baf-A1 elevated LC3II/LC3I, but Baf-A1-induced accumulation of LC3II was not further increased in the presence of SFN-Cys (Fig. 5i), indicating that SFN-Cys-mediated elevation of LC3II did not result from the formation of autophagosomes. CCCP is an uncoupling agent of mitochondrial proton carrier to destroy the mitochondrial membrane potential and induces mitophagy. TEM results showed that CCCP induced mitochondria disruption (Fig. 5j), while SFN-Cys induced more ruptured mitochondria and accumulated mitophagosomes coupled with apoptotic features in CCCP-treated cells than in sham group (Fig. 5j). Additionally, mitochondrial fusion protein Optic Atrophy 1 (OPA1) was significantly downregulated by SFN-Cys, while mitochondrial fission protein dynamin-related protein 1 was not affected (data not shown), suggesting SFN-Cys might inhibit mitochondrial fusion promoting mitochondrial fission, causing mitophagy inhibition (Fig. 5k). Likewise, SFN-Cys promoted the interaction of LC3II to lysosomal membrane-associated protein LAMP1 both in cytosol and mitochondria by co-immunoprecipitation (Fig. 5l). Further, we found that CCCP induced mitophagy leading to the elevated colocalization of LC3 to LAMP1 by confocal microscopy, while SFN-Cys exacerbated the colocalization of LC3 to LAMP1 in CCCP-treated cells, suggesting that SFN-Cys promoted the fusion of autophagosome to lysosome, which might be a cellular feedback against SFN-Cys stimulation (Fig. 5m). Chloroquine is a lysosomotropic agent. It accumulates inside the lysosomes causing inhibition of lysosomal enzymes that require an acidic pH and lysosomal protein degradation. Here, chloroquine significantly raised the conversion level of LC3I to II, while SFN-Cys did not further increase the conversion in chloroquine-treated cells (Fig. 5n), further suggesting that SFN-Cys impeded the autophagic flux and the degradation of autophagosome. However, we found that SFN-Cys downregulated lysosome-associated proteins or lysosomal proteases in the whole cells, such as Cathepsin D and tripeptidyl-peptidase 1 (TPP1), and downregulated Cathepsin 1 L in mitochondria by HPLC–MS/MS (Supplementary Table 2). Therefore, based on the results above, we concluded that SFN-Cys promoted the fusion of autophagosome to lysosome, but lowered the lysosomal degradation capacity, resulting in mitophagosome accumulation and mitophagy dysfunction.

### SFN-Cys inhibited migration and invasion by inhibiting mitophagy

**Fig. 6:**
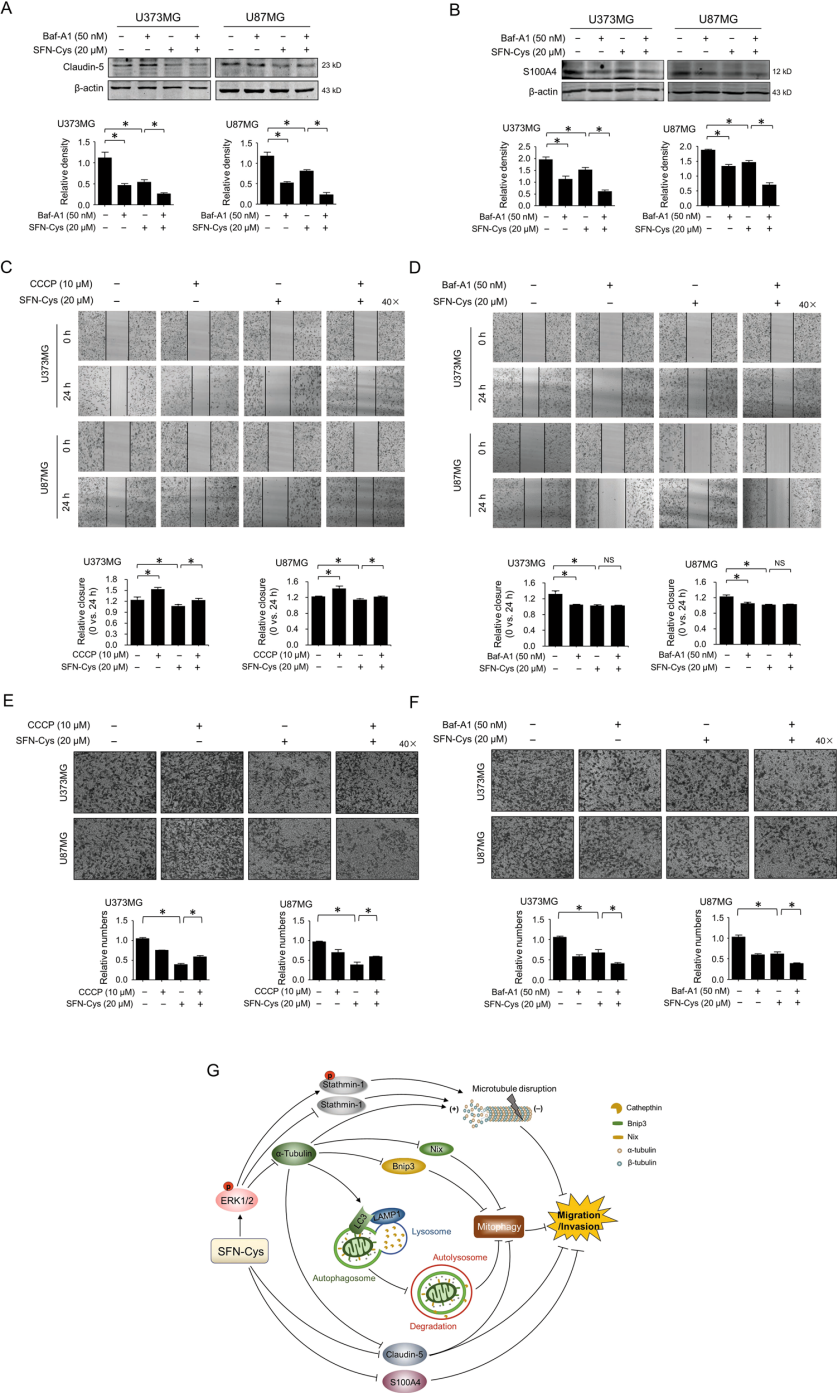
SFN-Cys inhibited migration and invasion by inhibiting mitophagy. The expression of Claudin-5 (**a**) or S100A4 (**b**) was detected by western blot with the treatment of 20 μM SFN-Cys and/or 50 nM Baf-A1 for 24 h. Scratch assay was done to evaluate the migration ability with the treatment with 20 μM SFN-Cys and/or 10 μM CCCP for 24 h (**c**) or with the treatment of 20 μM SFN-Cys and/or 50 nM Baf-A1 for 24 h (**d**). The invasion assay was done to evaluate the invasion ability with the treatment of 20 μM SFN-Cys and/or 10 μM CCCP for 24 h (**e**) or with the treatment of 20 μM SFN-Cys and/or 50 nM Baf-A1 for 24 h (**f**). **g** The proposed signaling map for SFN-Cys-inhibited migration and invasion in human glioblastoma. At least three independent experiments were performed. β-actin was used to be the loading control. *p < 0.05; NS no significance. Data were shown as means ± SD (*n* ≥ 3).

Here we found that the expression of Claudin-5 and S100A4 were downregulated in Baf-A1-treated cells, and SFN-Cys exacerbated the downregulation in Baf-A1 treated cells by western blot (Fig. 6a, b), suggesting that SFN-Cys downregulated Claudin-5 and S100A4 by inhibiting autophagy. Scratch assay results showed that the relative wound closure (0 h vs. 24 h) was elevated in CCCP-treated group indicating mitophagy might promote migration (Fig. 6c), while SFN-Cys treatment decreased the CCCP-upregulated migration in human GBM cells (Fig. 6c). Besides, the relative wound closure was decreased after Baf-A1 treatment (Fig. 6d), while SFN-Cys did not significantly promote Baf-A1-downregulated migration (Fig. 6d). Further invasion assay results also showed that SFN-Cys decreased the invasive cell numbers in GBM (Fig. 6e), while this result was reversed by CCCP (Fig. 6e). Baf-A1 decreased the invasive cell numbers, while this result was exacerbated by SFN-Cys (Fig. 6f). These data indicated that SFN-Cys inhibited cell migration and invasion by repressing mitophagy in GBM cells.

Altogether, we found that SFN-Cys decreased the invasion-associated proteins Claudin-5 and S100A4 leading to the inhibition of migration and invasion in GBM. Besides, SFN-Cys decreased α-tubulin and stathmin-1 by promoting ERK1/2 phosphorylation causing microtubule destruction, the downregulation of mitophagy receptor Bnip3-Nix pathway and the reduction of lysosomal hydrolytic capacity, contributing to the inhibition of mitochondria clearance as well as migration and invasion in GBM cells (Fig. 6g).

## Discussion

In the present study, we demonstrated that SFN-Cys inhibited invasion in GBM cells via inhibiting mitophagy and downregulating invasion-associated protein Claudin-5 and S100A4. Invasive process involves multiple signaling pathways; hundreds of proteins might play roles in the cascade. By HPLC–MS/MS analysis we noticed that S100A4 was a target of SFN-Cys in GBM. By GEPIA server, we coincidently found that these GBM patients with higher expression of S100A4 have lower survival. Studies showed that S100A4 was highly expressed in metastatic tumor cells and the expression of S100A4 played a role in regulating GBM cell aggressiveness^31^. S100A4 interacts with cytoskeletal proteins and enhances metastasis of several types of tumor cells^32^. Therefore, we thought that SFN-Cys might downregulate S100A4 and disturb the interaction of S100A4 to microtubules causing the decreasing of invasiveness in GBM. Claudin-5 in endothelial cells promotes vascular permeability and angiogenesis leading to metastasis in tumor cells^33^. Also, Claudin-5 regulates the permeability of blood–brain barrier by modulating the proliferation, migration, and permeability of human brain vascular endothelial cells, especially through the signaling pathway of cell adhesion^28^. Bioinformatics analysis showed an increased level of Claudin-5 in GBM tissue type. These indicated that Claudin-5 is also a key invasion-associated biomarker. Further, we uncovered that cell migration and invasion were decreased after knockdown of S100A4 and Claudin-5. Here, our results showed that SFN-Cys downregulated the expression of S100A4 and Claudin-5 via phosphorylated ERK1/2. Therefore, SFN-Cys inhibited migration and invasion by ERK1/2-mediated downregulation of S100A4 and Claudin-5.

Recently, we reported that SFN metabolites induced apoptosis and inhibited migration and invasion via causing microtubule disruption^7,13,25^; here we further determined that SFN-Cys inhibited the invasion in GBM by phosphorylated ERK1/2-mediated downregulation of Stathmin-1 and α-tubulin, and microtubule disruption. Stathmin-1 stimulated microtubule catastrophes leading to promotion of microtubule disassembly, while pStathmin-1 (Ser 25) blocked the role of Stathmin-1^34^. Our results showed that SFN-Cys downregulated Stathmin-1 and induced pStathmin-1 by activating ERK1/2, suggesting that SFN-Cys promoted the polymerization of microtubules. However, the double effects of SFN-Cys on both polymerization (by Stathmin-1) and depolymerization (by α-tubulin) of microtubules might result in a dynamic imbalance of microtubule, triggering microtubule disruption, and the inhibition of migration and invasion in GBM^13^.

Autophagy is identified as protective process that is involved in removing damaged organelles including mitochondria, peroxisomes, and endoplasmic reticulum to maintain the balance of cell normal function^35^. Likewise, autophagy plays an important role in regulating tumor cell motility and metastasis^24,36^. Even, autophagy promotes metastatic tumor recurrence; knockdown of autophagy regulators inhibits GBM migration and invasion^24^. We previously revealed that SFN metabolites inhibited autophagy leading to migration and invasion^7^. Autophagy promotes focal adhesion disassembly and cell motility of metastatic tumor cells through the direct interaction of Paxillin to LC3^37^. Notably, previous reports suggested that SFN-NAC blocked the fusion of autophagosome to lysosome leading to the increased level of LC3II/LC3I, LC3II accumulation, and autophagy inhibition in NSCLC^7,25^. Likewise, here SFN-Cys upregulated LC3II/LC3I but not change SQSTM1/p62 level in GBM cells, indicating SFN-Cys did not induce the formation of autophagosome. Then we found that SFN-Cys did not aggravate the upregulation of LC3II/LC3I in the present of Bafilomycin A1, suggesting that SFN-Cys-mediated elevation of LC3II/LC3I was not due to the formation of autophagosomes. Similarly, SFN-Cys did not aggravate the upregulation of LC3II/LC3I in the present of chloroquine, suggesting SFN-Cys might inhibit either the fusion of autophagosomes to lysosomes or lysosomal degradation capacity. Whereas both immunofluorescence staining and co-immunoprecipitation results supported that SFN-Cys enhanced the fusion of autophagosome to lysosome in GBM^38^. Luckily, we identified that SFN-Cys downregulated both the lysosomal cysteine proteases Cathepsin D and TPP1 in the whole cell and Cathepsin 1 L in mitochondria by HPLC–MS/MS analysis. Therefore, SFN-Cys might inhibit the hydrolytic capacity and trafficking of lysosomes, blocking the last autophagic flux, impeding autophagosome clearance, and causing the accumulation of autophagosomes in GBM^39^. Interestingly, these findings revealed a distinct SFN-Cys-regulated molecular mechanism in autophagy process compared to NSCLC. In NSCLC, SFN metabolites inhibited autophagic flux by blocking the fusion of mitophagosome to lysosome^7,25^. Studies reported that the expression of several proteins including conjugated LC3, BECN1, and LAMP related to autophagy is increased after injury^40^. Therefore, SFN-Cys-promoted the fusion of autophagosomes to lysosomes might be a feedback for cells against stimuli in GBM. More, we found the LC3II/LC3I was increased after knockdown of α-tubulin, suggesting that SFN-Cys might inhibit autophagy via inducing microtubule disruption in GBM. It was reported that autophagy was able to secrete some specific factors, such as interleukin-6, MMP-2, and WNT-5A, which was required for tumor cell invasion^41^. Therefore, based on our studies, it was clear that SFN-Cys promoted microtubule disruption and ultimately inhibited degradation of autolysosomes leading to the inhibition of migration and invasion in GBM.

Generally, hypoxia-induced autophagy is considered nonspecific. Mitophagy is a selective form of autophagy in which mitochondria are specifically targeted for autophagic degradation. Cell motility involves drastic structural changes, a process that demands high levels of energy and fully functional mitochondria whose quality and quantity are balanced by mitophagy^42^. Bnip3 is a proapoptotic atypical BH3-only protein that has been reported to induce apoptosis, necrosis, or autophagy relying on the type of stress and cellular context^43^. As a hallmark of mitophagy, upregulation of Bnip3 was associated with an increase of autophagy flux and mitochondrial protein degradation, as well as mitophagosome formation; loss of Bnip3 led to the accumulation of damaged mitochondria^44^. Bnip3 contributes to ischemia and reperfusion (I/R) injury which triggers a protective stress response with upregulation of autophagy and removal of damaged mitochondria^45^. Herein, we found that SFN-Cys decreased mitophagy protein Bnip3 and Nix, and disrupted mitochondria morphology, indicating SFN-Cys might also inhibit mitophagy and hindered the removal of damaged mitochondria. Mitochondria are the critical targets of hypoxia-induced autophagy, and mitochondria dynamics determine the energy supply to reorganize the cytoskeleton and maintain cell movement^46^. Likewise, Bnip3 is also a pro-migratory protein. Deletion of Bnip3 decreases the formation of lamellipodia and filopodia, and the migratory ability of melanoma cells^47^. In our studies, CCCP promoted migration and invasion, whereas Bafilomycin A1 decreased migration and invasion significantly, demonstrating that autophagy/mitophagy was related to migration and invasion. Additionally, Bafilomycin A1 also lowered the expression of invasion-related proteins S100A4 and Claudin-5. Therefore, according to these results, we speculated that the inhibition of autophagy might reduce migration and invasion. In the present study, SFN-Cys downregulated Bnip3 and Nix, inhibiting mitophagy and the clearance of damaged mitochondria, which might result in the inhibition of migration and invasion. Besides, microtubules facilitate autophagosome formation and fusion of autophagosomes to endosomes^48^. Several studies have proposed the direct regulatory effect of mitophagy on cellular migration via stress fibers such as F-actin and tubulin^42^. As we know mitochondrial membrane protein Bnip3 and Nix interact with microtubule lumen protein LC3, which is an important step in the recruitment of mitochondria to nascent autophagosomes, indicating microtubule is associated with mitophagy^49^. Additionally, we found that the expression of Bnip3 and Nix was downregulated after knockdown of α-tubulin indicating that SFN-Cys triggered microtubule disruption resulting in inhibition of Bnip3/Nix-mediated mitophagy. As such, these outcomes may offer a novel therapeutic strategy against migration and invasion in GBM.

## Supplementary information

Table S1

Table S2
