# Supplementary material for: Sulforaphane-cysteine inhibited migration and invasion via enhancing mitophagosome fusion to lysosome in human glioblastoma cells
Source: Cell Death Dis. 2020 Oct 1;11(9):819. doi: 10.1038/s41419-020-03024-5 (PMC7530759; doi:10.1038/s41419-020-03024-5)
Supplement: Supplementary file 1 — Table S1 [file 41419_2020_3024_MOESM1_ESM.docx]

Supplementary Table 1. The candidates of the screened targets of SFN-Cys from the total proteins in glioblastoma U87MG by HPLC-MS/MS.

|  | Entry | Gene names | Protein names | Expression |
| --- | --- | --- | --- | --- |
| Microtubule (and associated) proteins | Q9BQE3 | TUBA1C TUBA6 | Tubulin alpha-1C chain (Alpha-tubulin 6) (Tubulin alpha-6 chain) [Cleaved into: Detyrosinated tubulin alpha-1C chain] | ↓ |
|  | P68371 | TUBB4B TUBB2C | Tubulin beta-4B chain (Tubulin beta-2 chain) (Tubulin beta-2C chain) | ↓ |
|  | Q9H0B6 | KLC2 | Kinesin light chain 2 (KLC 2) | ↓ |
|  | Q3ZCM7 | TUBB8 | Tubulin beta-8 chain (Tubulin beta 8 class VIII) | ↓ |
|  | A6NHL2 | TUBAL3 | Tubulin alpha chain-like 3 | ↓ |
|  | Q9BSJ2 | TUBGCP2 GCP2 | Gamma-tubulin complex component 2 (GCP-2) (hGCP2) (Gamma-ring complex protein 103 kDa) (h103p) (hGrip103) (Spindle pole body protein Spc97 homolog) (hSpc97) | ↓ |
|  | P07437 | TUBB TUBB5 OK/SW-cl.56 | Tubulin beta chain (Tubulin beta-5 chain) | ↓ |
|  | Q71U36 | TUBA1A TUBA3 | Tubulin alpha-1A chain (Alpha-tubulin 3) (Tubulin B-alpha-1) (Tubulin alpha-3 chain) [Cleaved into: Detyrosinated tubulin alpha-1A chain] | ↓ |
|  | Q14166 | TTLL12 KIAA0153 | Tubulin--tyrosine ligase-like protein 12 (Inactive tubulin--tyrosine ligase-like protein 12) | ↓ |
|  | P68363 | TUBA1B | Tubulin alpha-1B chain (Alpha-tubulin ubiquitous) (Tubulin K-alpha-1) (Tubulin alpha-ubiquitous chain) [Cleaved into: Detyrosinated tubulin alpha-1B chain] | ↓ |
|  | P23258 | TUBG1 TUBG | Tubulin gamma-1 chain (Gamma-1-tubulin) (Gamma-tubulin complex component 1) (GCP-1) | ↓ |
|  | Q66K74 | MAP1S BPY2IP1 C19orf5 MAP8 VCY2IP1 | Microtubule-associated protein 1S (MAP-1S) (BPY2-interacting protein 1) (Microtubule-associated protein 8) (Variable charge Y chromosome 2-interacting protein 1) (VCY2-interacting protein 1) (VCY2IP-1) [Cleaved into: MAP1S heavy chain; MAP1S light chain] | ↓ |
|  | P16949 | STMN1 C1orf215 LAP18 OP18 | Stathmin (Leukemia-associated phosphoprotein p18) (Metablastin) (Oncoprotein 18) (Op18) (Phosphoprotein p19) (pp19) (Prosolin) (Protein Pr22) (pp17) | ↓ |
|  | Q9GZQ8 | MAP1LC3B MAP1ALC3 | Microtubule-associated proteins 1A/1B light chain 3B (Autophagy-related protein LC3 B) (Autophagy-related ubiquitin-like modifier LC3 B) (MAP1 light chain 3-like protein 2) (MAP1A/MAP1B light chain 3 B) (MAP1A/MAP1B LC3 B) (Microtubule-associated protein 1 light chain 3 beta) | ↑ |
| Autophagy/  Mitophagy | Q92609 | TBC1D5 KIAA0210 | TBC1 domain family member 5 | ↓ |
|  | Q9Y6I9 | TEX264 ZSIG11 UNQ337/PRO536 | Testis-expressed protein 264 (Putative secreted protein Zsig11) | ↓ |
|  | Q9H1Y0 | ATG5 APG5L ASP | Autophagy protein 5 (APG5-like) (Apoptosis-specific protein) | ↓ |
|  | Q66K74 | MAP1S BPY2IP1 C19orf5 MAP8 VCY2IP1 | Microtubule-associated protein 1S (MAP-1S) (BPY2-interacting protein 1) (Microtubule-associated protein 8) (Variable charge Y chromosome 2-interacting protein 1) (VCY2-interacting protein 1) (VCY2IP-1) [Cleaved into: MAP1S heavy chain; MAP1S light chain] | ↓ |
|  | Q13131 | PRKAA1 AMPK1 | 5'-AMP-activated protein kinase catalytic subunit alpha-1 (AMPK subunit alpha-1) (EC 2.7.11.1) (Acetyl-CoA carboxylase kinase) (ACACA kinase) (EC 2.7.11.27) (Hydroxymethylglutaryl-CoA reductase kinase) (HMGCR kinase) (EC 2.7.11.31) (Tau-protein kinase PRKAA1) (EC 2.7.11.26) | ↓ |
|  | Q16543 | CDC37 CDC37A | Hsp90 co-chaperone Cdc37 (Hsp90 chaperone protein kinase-targeting subunit) (p50Cdc37) [Cleaved into: Hsp90 co-chaperone Cdc37, N-terminally processed] | ↑ |
|  | Q9H444 | CHMP4B C20orf178 SHAX1 | Charged multivesicular body protein 4b (Chromatin-modifying protein 4b) (CHMP4b) (SNF7 homolog associated with Alix 1) (SNF7-2) (hSnf7-2) (Vacuolar protein sorting-associated protein 32-2) (Vps32-2) (hVps32-2) | ↑ |
|  | Q9GZQ8 | MAP1LC3B MAP1ALC3 | Microtubule-associated proteins 1A/1B light chain 3B (Autophagy-related protein LC3 B) (Autophagy-related ubiquitin-like modifier LC3 B) (MAP1 light chain 3-like protein 2) (MAP1A/MAP1B light chain 3 B) (MAP1A/MAP1B LC3 B) (Microtubule-associated protein 1 light chain 3 beta) | ↑ |
|  | O43504 | LAMTOR5 HBXIP XIP | Ragulator complex protein LAMTOR5 (Hepatitis B virus X-interacting protein) (HBV X-interacting protein) (HBX-interacting protein) (Late endosomal/lysosomal adaptor and MAPK and MTOR activator 5) | ↑ |
|  | Q9UI12 | ATP6V1H CGI-11 | V-type proton ATPase subunit H (V-ATPase subunit H) (Nef-binding protein 1) (NBP1) (Protein VMA13 homolog) (V-ATPase 50/57 kDa subunits) (Vacuolar proton pump subunit H) (Vacuolar proton pump subunit SFD) | ↑ |
|  | P36543 | ATP6V1E1 ATP6E ATP6E2 | V-type proton ATPase subunit E 1 (V-ATPase subunit E 1) (V-ATPase 31 kDa subunit) (p31) (Vacuolar proton pump subunit E 1) | ↑ |
|  | P60520 | GABARAPL2 FLC3A GEF2 | Gamma-aminobutyric acid receptor-associated protein-like 2 (GABA(A) receptor-associated protein-like 2) (Ganglioside expression factor 2) (GEF-2) (General protein transport factor p16) (Golgi-associated ATPase enhancer of 16 kDa) (GATE-16) (MAP1 light chain 3-related protein) | ↑ |
| Cell adhesion | Q92797 | SYMPK SPK | Symplekin | ↓ |
|  | P12111 | COL6A3 | Collagen alpha-3(VI) chain | ↓ |
|  | O14786 | NRP1 NRP VEGF165R | Neuropilin-1 (Vascular endothelial cell growth factor 165 receptor) (CD antigen CD304) | ↓ |
|  | P49757 | NUMB C14orf41 | Protein numb homolog (h-Numb) (Protein S171) | ↓ |
|  | O43294 | TGFB1I1 ARA55 | Transforming growth factor beta-1-induced transcript 1 protein (Androgen receptor coactivator 55 kDa protein) (Androgen receptor-associated protein of 55 kDa) (Hydrogen peroxide-inducible clone 5 protein) (Hic-5) | ↓ |
|  | P62140 | PPP1CB | Serine/threonine-protein phosphatase PP1-beta catalytic subunit (PP-1B) (PPP1CD) (EC 3.1.3.16) (EC 3.1.3.53) | ↓ |
|  | Q86W92 | PPFIBP1 KIAA1230 | Liprin-beta-1 (Protein tyrosine phosphatase receptor type f polypeptide-interacting protein-binding protein 1) (PTPRF-interacting protein-binding protein 1) (hSGT2) | ↓ |
|  | Q9Y5K6 | CD2AP | CD2-associated protein (Adapter protein CMS) (Cas ligand with multiple SH3 domains) | ↓ |
|  | Q6WCQ1 | MPRIP KIAA0864 MRIP RHOIP3 | Myosin phosphatase Rho-interacting protein (M-RIP) (Rho-interacting protein 3) (RIP3) (p116Rip) | ↓ |
|  | Q07020 | RPL18 | 60S ribosomal protein L18 (Large ribosomal subunit protein eL18) | ↓ |
|  | Q9BZE4 | GTPBP4 CRFG NOG1 | Nucleolar GTP-binding protein 1 (Chronic renal failure gene protein) (GTP-binding protein NGB) | ↓ |
|  | P62888 | RPL30 | 60S ribosomal protein L30 (Large ribosomal subunit protein eL30) | ↓ |
|  | P02751 | FN1 FN | Fibronectin (FN) (Cold-insoluble globulin) (CIG) [Cleaved into: Anastellin; Ugl-Y1; Ugl-Y2; Ugl-Y3] | ↓ |
|  | Q02952 | AKAP12 AKAP250 | A-kinase anchor protein 12 (AKAP-12) (A-kinase anchor protein 250 kDa) (AKAP 250) (Gravin) (Myasthenia gravis autoantigen) | ↓ |
|  | Q14161 | GIT2 KIAA0148 | ARF GTPase-activating protein GIT2 (ARF GAP GIT2) (Cool-interacting tyrosine-phosphorylated protein 2) (CAT-2) (CAT2) (G protein-coupled receptor kinase-interactor 2) (GRK-interacting protein 2) | ↓ |
|  | P05106 | ITGB3 GP3A | Integrin beta-3 (Platelet membrane glycoprotein IIIa) (GPIIIa) (CD antigen CD61) | ↓ |
|  | Q07157 | TJP1 ZO1 | Tight junction protein ZO-1 (Tight junction protein 1) (Zona occludens protein 1) (Zonula occludens protein 1) | ↑ |
|  | P48059 | LIMS1 PINCH PINCH1 | LIM and senescent cell antigen-like-containing domain protein 1 (Particularly interesting new Cys-His protein 1) (PINCH-1) (Renal carcinoma antigen NY-REN-48) | ↑ |
|  | O00592 | PODXL PCLP PCLP1 | Podocalyxin (GCTM-2 antigen) (Gp200) (Podocalyxin-like protein 1) (PC) (PCLP-1) | ↑ |
|  | Q9UH65 | SWAP70 KIAA0640 HSPC321 | Switch-associated protein 70 (SWAP-70) | ↑ |
| Migration/  Invasion | P30530 | AXL UFO | Tyrosine-protein kinase receptor UFO (EC 2.7.10.1) (AXL oncogene) | ↓ |
|  | O75326 | SEMA7A CD108 SEMAL | Semaphorin-7A (CDw108) (JMH blood group antigen) (John-Milton-Hargen human blood group Ag) (Semaphorin-K1) (Sema K1) (Semaphorin-L) (Sema L) (CD antigen CD108) | ↓ |
|  | Q93008 | USP9X DFFRX FAM USP9 | Probable ubiquitin carboxyl-terminal hydrolase FAF-X (EC 3.4.19.12) (Deubiquitinating enzyme FAF-X) (Fat facets in mammals) (hFAM) (Fat facets protein-related, X-linked) (Ubiquitin thioesterase FAF-X) (Ubiquitin-specific protease 9, X chromosome) (Ubiquitin-specific-processing protease FAF-X) | ↓ |
|  | Q9UGP4 | LIMD1 | LIM domain-containing protein 1 | ↓ |
|  | Q9NRY4 | ARHGAP35 GRF1 GRLF1 KIAA1722 P190A p190ARHOGAP | Rho GTPase-activating protein 35 (Glucocorticoid receptor DNA-binding factor 1) (Glucocorticoid receptor repression factor 1) (GRF-1) (Rho GAP p190A) (p190-A) | ↓ |
|  | Q15654 | TRIP6 OIP1 | Thyroid receptor-interacting protein 6 (TR-interacting protein 6) (TRIP-6) (Opa-interacting protein 1) (OIP-1) (Zyxin-related protein 1) (ZRP-1) | ↓ |
|  | O75044 | SRGAP2 ARHGAP34 FNBP2 KIAA0456 SRGAP2A | SLIT-ROBO Rho GTPase-activating protein 2 (srGAP2) (Formin-binding protein 2) (Rho GTPase-activating protein 34) | ↓ |
|  | Q08722 | CD47 MER6 | Leukocyte surface antigen CD47 (Antigenic surface determinant protein OA3) (Integrin-associated protein) (IAP) (Protein MER6) (CD antigen CD47) | ↓ |
|  | Q08209 | PPP3CA CALNA CNA | Serine/threonine-protein phosphatase 2B catalytic subunit alpha isoform (EC 3.1.3.16) (CAM-PRP catalytic subunit) (Calmodulin-dependent calcineurin A subunit alpha isoform) | ↓ |
|  | P31949 | S100A11 MLN70 S100C | Protein S100-A11 (Calgizzarin) (Metastatic lymph node gene 70 protein) (MLN 70) (Protein S100-C) (S100 calcium-binding protein A11) [Cleaved into: Protein S100-A11, N-terminally processed] | ↓ |
|  | P08253 | MMP2 CLG4A | 72 kDa type IV collagenase (EC 3.4.24.24) (72 kDa gelatinase) (Gelatinase A) (Matrix metalloproteinase-2) (MMP-2) (TBE-1) [Cleaved into: PEX] | ↓ |
|  | Q9BZF1 | OSBPL8 KIAA1451 ORP8 OSBP10 | Oxysterol-binding protein-related protein 8 (ORP-8) (OSBP-related protein 8) | ↑ |
|  | O00560 | SDCBP MDA9 SYCL | Syntenin-1 (Melanoma differentiation-associated protein 9) (MDA-9) (Pro-TGF-alpha cytoplasmic domain-interacting protein 18) (TACIP18) (Scaffold protein Pbp1) (Syndecan-binding protein 1) | ↑ |
|  | Q9Y2I1 | NISCH IRAS KIAA0975 | Nischarin (Imidazoline receptor 1) (I-1) (IR1) (Imidazoline receptor antisera-selected protein) (hIRAS) (Imidazoline-1 receptor) (I1R) (Imidazoline-1 receptor candidate protein) (I-1 receptor candidate protein) (I1R candidate protein) | ↑ |
