# Supplementary material for: Sulforaphane-cysteine inhibited migration and invasion via enhancing mitophagosome fusion to lysosome in human glioblastoma cells
Source: Cell Death Dis. 2020 Oct 1;11(9):819. doi: 10.1038/s41419-020-03024-5 (PMC7530759; doi:10.1038/s41419-020-03024-5)
Supplement: Supplementary file 2 — Table S2 [file 41419_2020_3024_MOESM2_ESM.docx]

Supplementary Table 2. The identification of lysosomal associated proteins regulated by SFN-Cys in the whole-cell or in mitochondria by HPLC-MS/MS.

|  | Entry | Gene names | Protein names | Expression |
| --- | --- | --- | --- | --- |
| Lysosomal associated proteins in the whole cells | P42892 | ECE1 | Endothelin-converting enzyme 1 (ECE-1) (EC 3.4.24.71) | ↓ |
|  | O14773 | TPP1 CLN2 GIG1 UNQ267/PRO304 | Tripeptidyl-peptidase 1 (TPP-1) (EC 3.4.14.9) (Cell growth-inhibiting gene 1 protein) (Lysosomal pepstatin-insensitive protease) (LPIC) (Tripeptidyl aminopeptidase) (Tripeptidyl-peptidase I) (TPP-I) | ↓ |
|  | Q9BZG1 | RAB34 RAB39 RAH | Ras-related protein Rab-34 (Ras-related protein Rab-39) (Ras-related protein Rah) | ↓ |
|  | Q15165 | PON2 | Serum paraoxonase/arylesterase 2 (PON 2) (EC 3.1.1.2) (EC 3.1.1.81) (Aromatic esterase 2) (A-esterase 2) (Serum aryldialkylphosphatase 2) | ↓ |
|  | P50897 | PPT1 CLN1 PPT | Palmitoyl-protein thioesterase 1 (PPT-1) (EC 3.1.2.22) (Palmitoyl-protein hydrolase 1) | ↓ |
|  | Q86VS8 | HOOK3 | Protein Hook homolog 3 (h-hook3) (hHK3) | ↓ |
|  | Q9HD26 | GOPC CAL FIG | Golgi-associated PDZ and coiled-coil motif-containing protein (CFTR-associated ligand) (Fused in glioblastoma) (PDZ protein interacting specifically with TC10) (PIST) | ↓ |
|  | Q3ZAQ7 | VMA21 MEAX XMEA | Vacuolar ATPase assembly integral membrane protein VMA21 (Myopathy with excessive autophagy protein) | ↓ |
|  | P07339 | CTSD CPSD | Cathepsin D (EC 3.4.23.5) [Cleaved into: Cathepsin D light chain; Cathepsin D heavy chain] | ↓ |
|  | P46976 | GYG1 GYG | Glycogenin-1 (GN-1) (GN1) (EC 2.4.1.186) | ↑ |
|  | Q9BXS5 | AP1M1 CLTNM | AP-1 complex subunit mu-1 (AP-mu chain family member mu1A) (Adaptor protein complex AP-1 subunit mu-1) (Adaptor-related protein complex 1 subunit mu-1) (Clathrin assembly protein complex 1 mu-1 medium chain 1) (Clathrin coat assembly protein AP47) (Clathrin coat-associated protein AP47) (Golgi adaptor HA1/AP1 adaptin mu-1 subunit) (Mu-adaptin 1) (Mu1A-adaptin) | ↑ |
|  | O94759 | TRPM2 EREG1 KNP3 LTRPC2 TRPC7 | Transient receptor potential cation channel subfamily M member 2 (Estrogen-responsive element-associated gene 1 protein) (Long transient receptor potential channel 2) (LTrpC-2) (LTrpC2) (Transient receptor potential channel 7) (TrpC7) (Transient receptor potential melastatin 2) | ↑ |
|  | Q99519 | NEU1 NANH | Sialidase-1 (EC 3.2.1.18) (Acetylneuraminyl hydrolase) (G9 sialidase) (Lysosomal sialidase) (N-acetyl-alpha-neuraminidase 1) | ↑ |
|  | P07711 | CTSL CTSL1 | Cathepsin L1 (EC 3.4.22.15) (Cathepsin L) (Major excreted protein) (MEP) [Cleaved into: Cathepsin L1 heavy chain; Cathepsin L1 light chain] | ↑ |
|  | Q9NVJ2 | ARL8B ARL10C GIE1 | ADP-ribosylation factor-like protein 8B (ADP-ribosylation factor-like protein 10C) (Novel small G protein indispensable for equal chromosome segregation 1) | ↑ |
| Lysosomal associated proteins in mitochondria | Q86VS8 | HOOK3 | Protein Hook homolog 3 (h-hook3) (hHK3) | ↓ |
|  | Q9NX57 | RAB20 | Ras-related protein Rab-20 | ↓ |
|  | Q9BV23 | ABHD6 | Monoacylglycerol lipase ABHD6 (EC 3.1.1.23) (2-arachidonoylglycerol hydrolase) (Abhydrolase domain-containing protein 6) | ↓ |
|  | O15321 | TM9SF1 | Transmembrane 9 superfamily member 1 (MP70 protein family member) (hMP70) | ↓ |
|  | P15289 | ARSA | Arylsulfatase A (ASA) (EC 3.1.6.8) (Cerebroside-sulfatase) [Cleaved into: Arylsulfatase A component B; Arylsulfatase A component C] | ↓ |
|  | P07711 | CTSL CTSL1 | Cathepsin L1 (EC 3.4.22.15) (Cathepsin L) (Major excreted protein) (MEP) [Cleaved into: Cathepsin L1 heavy chain; Cathepsin L1 light chain] | ↓ |
|  | P11279 | LAMP1 | Lysosome-associated membrane glycoprotein 1 (LAMP-1) (Lysosome-associated membrane protein 1) (CD107 antigen-like family member A) (CD antigen CD107a) | ↑ |
|  | Q6IAA8 | LAMTOR1 C11orf59 PDRO PP7157 | Ragulator complex protein LAMTOR1 (Late endosomal/lysosomal adaptor and MAPK and MTOR activator 1) (Lipid raft adaptor protein p18) (Protein associated with DRMs and endosomes) (p27Kip1-releasing factor from RhoA) (p27RF-Rho) | ↑ |
